# Genetic Determinants and Clonal Composition of Levofloxacin-Resistant *Streptococcus agalactiae* Isolates from Bulgaria

**DOI:** 10.3390/antibiotics14111121

**Published:** 2025-11-07

**Authors:** Vasil S. Boyanov, Alexandra S. Alexandrova, Raina T. Gergova

**Affiliations:** Department of Medical Microbiology, Medical Faculty, Medical University of Sofia, Zdrave Str. 2, 1431 Sofia, Bulgaria; alexandrova_sa@medfac.mu-sofia.bg

**Keywords:** *S. agalactiae*, GBS, fluoroquinolones, levofloxacin resistance, missense mutations, QRDR, serotypes, multi-locus sequence typing, MLST

## Abstract

**Background**: Levofloxacin is a broad-spectrum third-generation fluoroquinolone with bactericidal activity against *Streptococcus* species. We aimed to investigate the susceptibility rates of levofloxacin, the genetic determinants contributing to resistance, the serotype distribution, and the population structure of levofloxacin-resistant *Streptococcus agalactiae* (GBS) isolates. **Methods**: Antibiotic susceptibility testing was conducted according to the EUCAST criteria. PCR-serotyping, determination of mutations in the quinolone resistance-determining regions (QRDRs), and multi-locus sequence typing (MLST) were performed on all levofloxacin-resistant strains. **Results**: Among the 328 GBS isolates, 11.9% exhibited resistance to levofloxacin. We categorized the samples into two main groups: vaginal (64.1%) and extra-vaginal. The latter was further subdivided into invasive (10.3%) and non-invasive (25.6%) ones. The most common serotypes identified were V (30.8%) and III (25.6%). All examined resistant strains possessed missense mutations in the QRDR of *parC* (Ser79Phe/Tyr and Asp83Asn), whereas 59.0% of them exhibited additional mutation in *gyrA* (Ser81Leu and Glu85Lys/Ala). The MLST results disclosed six clonal complexes: CC19(64.1%), followed by CC1 (10.3%), CC452 (7.7%), and CC459 (5.1%), and CC12 and CC23, represented by single strains. **Conclusions**: We observed a growing resistance to fluoroquinolones that appears to exceed the average in Europe. More than half of the isolates exhibited the accumulation of mutations within the QRDRs. Rigorous monitoring is needed to prevent the emergence of MDR GBS and preserve the effectiveness of the newer generations of fluoroquinolones.

## 1. Introduction

*Streptococcus agalactiae* (group B Streptococcus, GBS) is a beta-hemolytic opportunistic pathogen that causes a wide range of invasive diseases in individuals with compromised immune system and frequently manifests in conjunction with chronic non-infectious comorbidities [1,2]. The ability of GBS to cause disease is associated with various virulence factors. The capsular polysaccharide (CP) is considered one of the most important, as it helps in evading the immune response and is crucial for the bacteria’s survival. There are ten identified serotypes (Ia, Ib, II–IX), classified according to the differences in the chemical structure and antigenicity of the CP [3]. GBS is associated with neonatal infections and has been recognized as a significant contributor to invasive streptococcal diseases in non-pregnant adult populations [4,5]. This encompasses SSTI, which includes conditions such as abscess, cellulitis, and diabetic foot infections, along with bacteremia and infections of the urogenital tract, as well as respiratory tract infections and meningitis. The majority of the diseases presented align with the indications for treatment with fluoroquinolones [6,7]. The mechanisms that lead to antibiotic resistance in GBS include genetic mutations, horizontal gene transfer, and the acquisition of resistance genes from different bacterial species. Furthermore, the differences in resistance rates may be associated with local prescribing practices and public health policies [8].

Levofloxacin is categorized as a third-generation fluoroquinolone and is recognized as an essential medicine by the World Health Organization [9]. It is a chiral molecule and represents the pure (−)-(*S*)-enantiomer of the racemic second-generation ofloxacin. Levofloxacin exhibits superior antibacterial efficacy against both Gram-positive and Gram-negative bacteria when compared to the (*R*)-enantiomer [10,11,12]. It is a broad-spectrum fluoroquinolone antibiotic that is effective against Gram-positive, Gram-negative, and atypical bacteria, as well as a selected activity against some anaerobic and microaerophilic bacteria [13,14]. Levofloxacin demonstrates significant activity against various species within the *Streptococcus* genus and is used for the treatment of respiratory and urogenital tract infections. This is attributed to its capacity to achieve high intrapulmonary concentrations and its excretion in urine as an unchanged active drug [7,15]. This antibiotic is also indicated for the treatment of skin and soft tissue infections (SSTIs) as well as severe cases of anthrax [16,17].

Levofloxacin exhibits an improved safety profile compared to older generations of fluoroquinolones including a reduced probability of central nervous system adverse effects and limited skin reactions. Furthermore, it demonstrates a lower incidence of QT interval prolongation and anaphylactic reactions in comparison to the fourth-generation fluroquinolone moxifloxacin. However, levofloxacin poses one of the highest risks for tendinopathy or tendon rupture, as well as the exacerbation of myasthenia gravis [18]. Similar to other quinolones, levofloxacin is not advised for use in pregnant individuals and children due to potential toxicity. It should only be administered when no alternatives are available, and the benefit must outweigh the risk [19,20].

The mechanism of action of quinolones involves the inhibition of the catalytic functions of bacterial topoisomerase enzymes, DNA gyrase, and topoisomerase IV, which produce negative supercoils and facilitate the decatenation of DNA, respectively. This leads to an increased concentration of drug–enzyme–DNA cleavage complexes, converting them into cellular toxins that induce irreversible breaks in DNA and chromosome fragmentation. This ultimately inhibits bacterial DNA replication and transcription, resulting in a bactericidal effect [21,22].

The main mechanism of resistance to quinolones involves mutations in the quinolone resistance-determining regions (QRDRs) of the genes that encode topoisomerases (*gyrA* and *gyrB* for DNA gyrase; *parC* and *parE* for topoisomerase IV). Less frequently, resistance develops as a result of mutations in efflux regulatory genes, which cause the active removal of drugs from the cytoplasm to the cell exterior [15,23]. However, due to its hydrophobic chemical structure, levofloxacin is not an appropriate substrate for efflux pumps. Notably, the use of reserpine, which acts as an inhibitor of active efflux, did not change the minimum inhibitory concentration (MIC) of this antibiotic, excluding the involvement of this mechanism in the interpretation of levofloxacin non-susceptibility [23,24].

Missense mutations in the QRDRs that result in amino acid substitutions and determine the resistance to fluoroquinolones in GBS, were described in GyrA at positions Ser-81 and Glu-85, as well as in ParC at positions Ser-79 and Asp-83. A considerable number of silent mutations that do not affect the phenotype have also been identified [25,26,27,28]. Mutations in *gyrB* and *parE* were identified in older generations of quinolones; however, there is no evidence suggesting that these mutations are associated with resistance to levofloxacin [27,29,30].

One of the most commonly used techniques for the epidemiological characterization of bacterial species is multi-locus sequence typing (MLST). The advantages of this method include its high efficiency, productivity, and discriminative power, establishing it as a reference method for the epidemiological typing of GBS [31,32,33]. Identifying GBS isolates that exhibit resistance to fluoroquinolones and evaluating their epidemiological prevalence in relation to sequence types (STs), clonal complexes (CCs), and serotype distribution provides crucial insights for monitoring the spread of multidrug-resistant (MDR) isolates [5,34].

We aimed to investigate the prevalence of levofloxacin resistance in GBS isolates obtained from Bulgarian patients during the period of 2021–2025, analyzing serotype distribution, identifying mutations in the QRDR, and examining clonality within the studied population. This study represents the first population genetic structure conducted on fluoroquinolone-resistant GBS strains in Bulgaria, providing valuable insights for their surveillance.

## 2. Results

### 2.1. Studied Population

During routine diagnostics, we isolated 39 levofloxacin-resistant GBS strains (age range (AR): 17–88 years; mean age (MA): 42.2 years of age from a total of 328 isolates examined. We categorized the samples into two groups according to their source. The first group consisted of vaginal samples (n = 25; AR: 17–67; MA: 37.4 years) collected from pregnant (52.0%) and non-pregnant women with confirmed genital infection with GBS as either a co-infectious agent in bacterial vaginosis or as a leading pathogen in aerobic vaginitis. Coexisting conditions in this group were psoriasis, cervical carcinoma, and diabetes type 2. The second group contained 14 extra-vaginal samples divided according to their source into two subgroups. The invasive subgroup (AR: 18–67; MA: 45.5 years) was collected from sterile sites such as blood culture, soft tissue wound aspirates, and tracheal aspirates. Notable comorbidities included type 1 diabetes, chronic obstructive pulmonary disease, and arterial thrombosis of lower limbs. In the non-invasive subgroup (AR: 22–88; MA: 52.9 years), which included urine and ejaculate samples, type 2 diabetes, prostate hypertrophy, hydronephrosis, and chronic kidney disease were observed.

### 2.2. Levofloxacin Susceptibility

**Figure 1:**
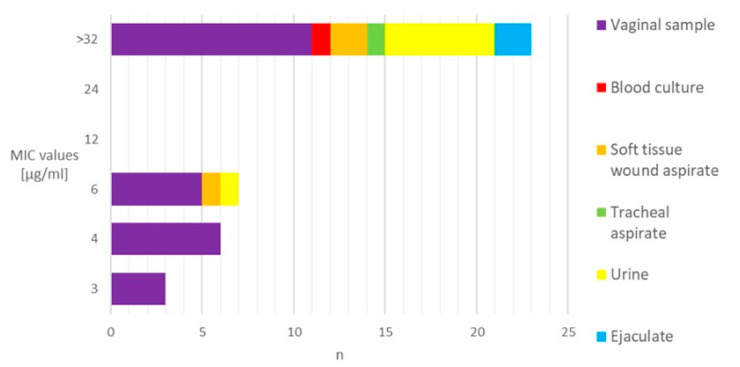
Distribution of levofloxacin-resistant GBS strains based on the MIC values and sample sources. MIC—minimum inhibitory concentration. n—number of strains.

Among the 328 GBS strains examined, 39 (11.9%) exhibited resistance to levofloxacin. The MIC values of the resistant isolates, when presented in relation to the material source, indicated that the majority of them (59.0%) demonstrated resistance levels exceeding 32 μg/mL. The vaginal samples predominantly showed a low level of resistance (<8 μg/mL), whereas the majority of the extra-vaginal samples (85.7%) exhibited high MIC values (Figure 1).

### 2.3. Serotyping

Six out of ten GBS serotypes were identified among resistant strains. The predominant serotypes were V and III, accounting for 56.4%, while the remaining serotypes (II, IV, VI, and Ia) represented 35.9%, respectively. Three isolates were non-typable. Serotype Ia was significantly more common among susceptible isolates (*p* = 0.0002) (Table 1). Furthermore, a statistical significance was noted in the distribution of serotypes III and V when comparing isolates with an MIC > 32 μg/mL to those with an MIC < 32 μg/mL (*p* <0.05) (Table S1).

### 2.4. Mutations in Genes Encoding Topoisomerases

**Figure 2:**
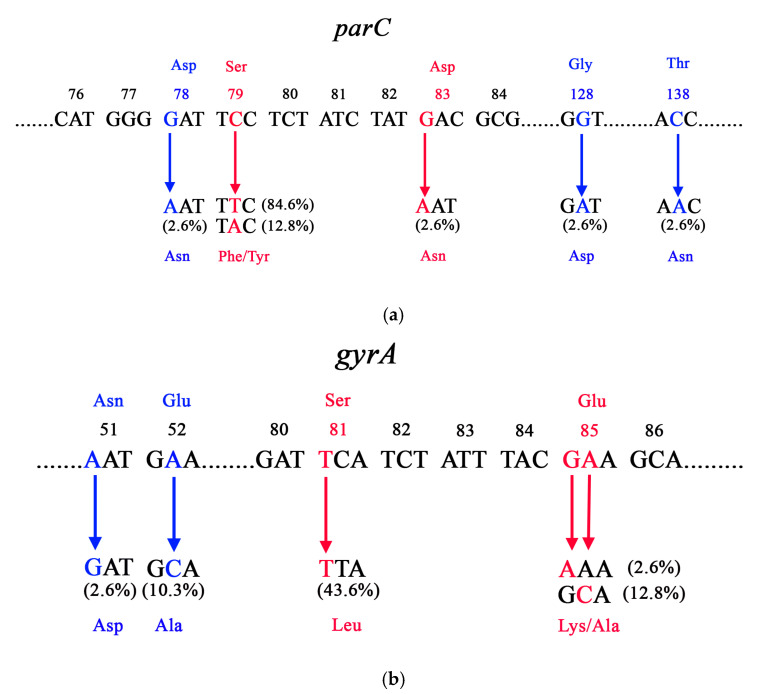
Missense mutations in the (**a**) *parC* and (**b**) *gyrB* genes among levofloxacin-resistant GBS isolates. Red color substitutions indicate mutations in QRDRs, whereas blue color substitutions represent additional mutations located outside this region. Asp—aspartic acid, Ser–serine, Gly—glycine, Asn—asparagine, Phe—phenylalanine, Tyr—tyrosine, Thr—threonine, Glu—glutamic acid, Ala—alanine, Leu—leucine, Lys—lysine.

All examined strains harbored missense mutations in the QRDR region of *parC*, with Ser79Phe being the most prevalent, followed by Ser79Tyr and Asp83Asn. Additionally, three isolates possessed mutations leading to amino acid substitutions at positions 78, 128, and 138 when compared with the GBS reference strains (GTC 1234 and GTC 1966) using BLAST 2.16.0, available at NCBI (http://www.ncbi.nlm.nih.gov/BLAST, accessed on 1 September 2025) (Figure 2a). Regarding *gyrA*, 59.0% of the isolates harbored missense mutations in the QRDR (Ser81Leu, Glu85Lys/Ala), while nucleotide changes in amino acids at positions 51 and 52 were noted in five isolates (Figure 2b). Silent mutations in *parC* were identified in all tested strains, whereas only a single isolate exhibited this type of mutation in *gyrA* (Table S2).

### 2.5. Multi-Locus Sequence Typing (MLST)

**Figure 3:**
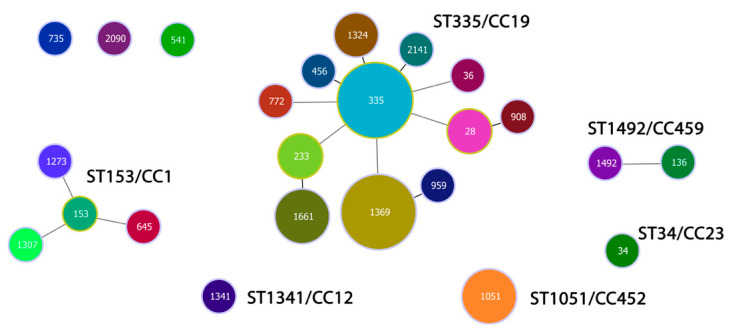
Population snapshot of 39 levofloxacin-resistant GBS isolates recovered in Bulgaria for a period (2021–2025). Each sequence type (ST) was marked in different colors. The size of the circles corresponds to the number of bacterial isolates. The length and thickness of the lines indicate the genetic distance between the isolates. The single locus variants (SLVs) were linked with shorter, bold lines. The double locus variants (DLVs) were connected by longer, grey lines.

We identified six CCs among the studied population of 39 levofloxacin-resistant strains: CC1, CC12, CC19, CC23, CC452, and CC459. They accounted for a total of 92.3% of the isolates. The closely related genotypes were grouped in CCs named after their ancestor and the reference MLST clones to which they belonged. We selected single (SLVs) and double (DLVs) loci variants to establish the clonal structure of the population, ensuring a clearer understanding of its genetic composition (Figure 3). SLVs refer to single locus variants, where the isolates differ in their allelic profiles based on seven housekeeping genes at one gene locus. DLVs, or double locus variants, indicate that the isolates differ in their allelic profiles based on seven housekeeping genes at two gene loci. CC19 was the most common, comprising 25 strains distributed in 12 STs (64.1%). The predominant founder and the earlier discovered type was ST335 (20.0%). Its SLVs were ST456, ST1324, and ST2141 (16.0%). The DLVs of ST335 (44.0%) were represented by ST772, ST36, ST233, ST1369, and ST28. Genotypes ST233, ST1369, and ST28 showed relatedness to other corresponding SLVs: ST1661, ST959, and ST908, respectively. CC1 was the second most distributed (10.3%), consisting of the main ST153 and its DLVs ST645, ST1273, and ST1307. CC459 (5.1%) involved DLVs ST136 and ST1492, represented by single strains. CC452 (7.7%) comprised three isolates of ST1051. ST1341 and ST34 showed relatedness to reference MLST clones CC12 and CC23, respectively. Three strains among the studied population of levofloxacin-resistant isolates did not show relatedness to any reference clone from the MLST database (Table S3).

Regarding the distribution of CCs according to the source of material, no statistical significance was observed in the vaginal and extra-vaginal samples (*p* > 0.05) (Table 2).

**Figure 4:**
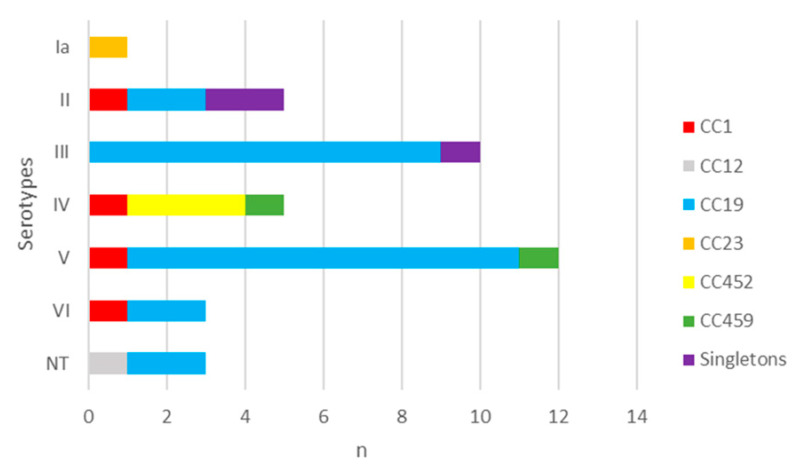
Distribution of serotypes in levofloxacin-resistant GBS strains according to clonal complexes. n—number of strains. I–IV—GBS serotypes. NT—non-typeable. CC—clonal complex.

The distribution of CCs by serotypes disclosed that CC1 consisted of four isolates of different serotypes. In the predominant CC19, which included 25 isolates, serotypes V (40.0%) and III (36.0%) were the most common. CC452 was represented by three strains of serotype IV, CC459 by two isolates of serotype IV and V, while the singletons were of serotype II and III (Figure 4). There was no statistical significance in the distribution of serotypes among strains from the most prevalent CC19 when compared to the other CCs (*p* > 0.05) (Table S4).

## 3. Discussion

In recent years, GBS has been established as an emerging opportunistic pathogen due to its ability to cause various invasive infections with different locations [1]. Currently, most cases of invasive GBS disease occur in adults aged 65 and above, particularly those with chronic non-infectious comorbidities [35]. Furthermore, the number of MDR GBS isolates has been increasing, causing growing concern [7,36]. This observation, along with the changes in clonal composition and the emergence of new, more virulent CCs, may result in further alternations in the incidence and severity of diseases within the population [37,38]. Epidemiological typing and its relation to the spread of antibiotic resistance are essential in GBS surveillance, pointing to their significance especially regarding immunocompromised patients, as this pathogen poses an increased risk of severe, life-threatening condition [8,39].

Beta-lactam antibiotics are the first-line medications for the prevention and treatment of GBS infections due to their widespread susceptibility and safety profile. However, reports of resistant strains and occurrences of allergic reactions may require the use of second-line alternatives, such as macrolides, lincosamides, or fluoroquinolones [1,40]. In Bulgaria, high resistance rates to macrolides and clindamycin have been reported [41]. Subsequently, the rise in fluoroquinolone resistance could affect treatment options and complicate empiric regiments in the case of severe infections [7]. Furthermore, the extensive use and numerous indications of fluoroquinolones in medicine presumptively lead to the selection of strains with more than one mutation in the genes that encode topoisomerases [12,42,43]. This results in an increased prevalence of MDR GBS and contributes to the emergence of strains that exhibit resistance to novel classes of fluoroquinolones, even before their implementation in therapeutic practices [42,44,45]. Therefore, the limited options for the management of patients with penicillin allergies, increased risk of treatment failures in cases of severe GBS infections, and the emergence and spread of MDR clones highlights the significance of fluoroquinolone non-susceptibility surveillance.

The prevalence of levofloxacin resistance in GBS throughout Europe indicated that the lowest resistance rates (below 5.0%) have been observed in Portugal and France, whereas Italy and Romania reported approximately 10.0%, and the highest rate was found in Turkey at 28.8% [46,47,48,49,50]. In North America, the prevalence of non-susceptible strains consistently remained below 2.5% across three consecutive studies conducted in the USA. In Canada, a rate of 12.4% was documented, while in South American countries, it varied between 5.0% and 11.0% [29,51,52,53]. In Africa, a research study encompassing 21 countries indicated an overall fluoroquinolone resistance rate of 24.6% [54]. In the analysis of data obtained from Asia, it was noted that this region displays the highest rates of fluoroquinolone resistance compared to other parts of the world, reaching 72.9% [55,56,57]. In the current study, 11.9% of the GBS strains examined exhibited resistance to levofloxacin. In comparison to the non-susceptibility of levofloxacin with the earlier study conducted in Bulgaria, we observed a 1.6% rise in resistance [58]. This result demonstrates a trend of increasing resistance that exceeds the reported values of the majority of countries in Europe.

Studies indicate that individual mutations in the genes responsible for encoding topoisomerase IV and DNA gyrase (*parC* and *gyrA*) result in low-level resistance to levofloxacin, while MIC values exceeding 32 μg/mL are caused by a combination of nucleotide changes in the sequence of the QRDRs of both enzymes [59,60]. In the current study, mutations in the QRDRs of *parC* were identified in all levofloxacin-resistant GBS isolates. Furthermore, 59.0% of these isolates exhibited additional missense mutations in the QRDRs of *gyrA*, which corresponded with the number of isolates demonstrating MICs greater than 32 μg/mL, in accordance with the aforementioned data. Moreover, serotypes III and V were significantly distributed among isolates with MICs greater than 32 μg/mL. These strains exhibited accumulation of mutations in the QRDR and were associated with serotypes being more prevalent in invasive diseases. This indicates the selection of the most virulent serotypes in fluoroquinolone-resistant strains [42,61]. According to the data, the presence of multiple mutations leads to the development of non-susceptibility to newer classes of fluoroquinolones, which exhibit similar resistance mechanisms and may potentially hinder their use in the future [42,44].

In *parC* and *gyrA*, alongside the most common mutations that lead to resistance (Ser79Phe/Tyr and Ser81Leu, respectively), we identified additional mutations that cause amino acid substitutions [26,27]. According to the data, the mutations Asp78Asn and Asp83Asn in *parC* were initially discovered in *S. pneumoniae*. However, subsequent findings revealed that the substitution at position 83 is significant in GBS and leads to resistance [60,62]. This finding is supported by the current study, which identified a strain that possessed only this missense mutation and demonstrated a low MIC level (4 μg/mL). The other amino acid substitution (Asn78Asp) was identified alongside Ser79Phe in an isolate with an MIC value 3 μg/mL. This observation, along with the absence of substantial evidence in the literature indicating the involvement of this mutation in resistance, suggests that it is probably not significant in relation to fluoroquinolone resistance in GBS [60,62]. Regarding *gyrA*, five strains demonstrating non-susceptibility were identified, each possessing a single mutation in this gene that results in an amino acid substitution at position 85 (Glu85Ala). This mutation has been documented to date in *S. pyogenes*, *S. pneumoniae*, and in one report related to GBS from Miró et al. (2006) [63,64,65]. In pneumococci, this mutation has demonstrated a correlation with increased MICs for fluoroquinolones [63]. This mutation was detected in isolates showing MIC >32 μg/mL, which were associated with the presence of amino acid substitutions in the QRDRs of both topoisomerase enzymes. Alongside the absence of any other identified mutation for *gyrA* in these sequences from the examined strains, it can be concluded that this amino acid substitution at position 85 (GAA→GCA) contributes to resistance. This confirms the findings made by the Brazilian authors [64]. Additionally, in one isolate, we identified a mutation at the same amino acid position, where glutamate is replaced by lysine (Glu85Lys, GAA→AAA). This mutation has been documented as a contributing factor to resistance against fluoroquinolones, thereby categorizing the discussed sequence (GAA) as a part of QRDRs [27]. We also discovered three more missense mutations in *parC* and two in *gyrA*: Asp78Asn, Gly128Asp, and Thr138Asn, as well as Asn51Asp and Glu52Ala, respectively. Due to insufficient data supporting their significance in fluoroquinolone resistance, they remain a subject for further investigation.

In the analysis of data derived from global studies on circulating CCs of GBS, it was found that five CCs are predominantly associated with human diseases: CC1, CC10, CC17, CC19, and CC23 [32]. Moreover, certain CCs are more common in particular serotypes and clinical manifestations. CC17 is associated with serotype III, the most prevalent serotype related to the onset of invasive neonatal diseases. The four remaining CCs are recognized as prevalent colonizers of the vaginal mucosa, demonstrating a reduced invasive potential. CC1 is associated with serotype V and is derived from infections in adult non-pregnant individuals, while CC23 is linked to serotype Ia [32,61,66].

In the present study, six CCs (CC1, CC12, CC19, CC23, CC452, and CC459) were identified among levofloxacin-resistant GBS strains. CC19 contained the highest number of strains (64.1%), with serotypes V and III being the most frequently observed among them, followed by CC1 (10.3%, no prevailing serotype), CC452 (7.7%, IV), CC459 (5.1%, IV and V), CC12 (2.6%, NT), and CC23(2.6%, Ia). In Europe, an analysis of 666 isolates from pregnant women with culture-positive GBS across eight countries revealed a prevalence of CC19 (23.6%) [67]. Comparable data were obtained in the UK, while in France, CC1 exhibited the highest frequency in two distinct studies [68,69,70]. Additionally, a report from Italy provided information regarding the less common CC452, predominantly identified in serotype IV [71]. Regarding neonatal infections, all studies indicate that CC17 was the most prevalent, followed by CC19 or CC23 [66,72,73,74]. A limited number of studies documenting MLST on levofloxacin-resistant strains in Europe have been published in the literature. In two studies conducted on fluoroquinolone-resistant GBS strains in Europe, from Italy (n = 8) and France (n = 66), a frequency of CCs was detected, with CC19 (mainly from serotypes V and III) prevailing in over 50% of the studied isolates [60,75]. The information provided regarding the GBS CCs in Europe, encompassing both the commonly circulating and levofloxacin-resistant strains, corresponds with our study, although there were slight variations in the dominant CCs and the serotypes that were most commonly reported within them.

In both North and South America, the majority of GBS strains were identified as CC23 (with serotype Ia being the most common), whereas 10% of the examined strains were categorized as CC452 and CC459 [52,53,76,77]. Similar results were recorded in Australia and Botswana [78,79]. In contrast, in other African studies, CC1 serotype V was identified as the most prevalent [80]. In Asia, CC1 was the predominant in Indonesia, Saudi Arabia, and South Korea, while CC19 serotype III was prevalent in Iran and China [8,33,57,81,82,83,84,85]. Due to the high levels of resistance to fluoroquinolones, the majority of data regarding resistant strains analyzed through MLST were mainly derived from Asian countries [7]. In China (n = 138), the most common CCs among levofloxacin-resistant GBS were CC19 (III) and CC10 (Ib); in Taiwan (n = 88), the predominant ones were CC19 (III) and CC1 (IV); in Japan (n = 76), leading were CC1 (V) and CC19 (III); while in South Korea (n = 16), the majority consisted of CC10 [28,86,87,88]. Certain studies indicated that CC19 was the most prevalent among resistant isolates, especially in China, where it exhibited a notable predominance, in concordance with the findings of the present study. The distribution of serotypes was influenced by the selection of invasive agents in most of the reported studies, resulting in a predominance of serotypes III and Ia. Serotype Ib was primarily found in Asia and North America, which probably explains the higher prevalence of CC12, often associated with this serotype, in these regions [89,90]. Regarding the rare CC452, we found that three strains of this CC were identified as serotype IV, which supports the studies that were analyzed [71,85,88].

Data suggest that CC19 is associated with an increased risk of invasive diseases and may act as a reservoir for non-vaccine strains as well as determinants of antimicrobial resistance [70,91]. Furthermore, GBS contains multiple mobile genetic elements in its genome that are associated with clonal expansion across the GBS population. These carriers facilitate the transfer of virulence and resistance genes, which may serve as genetic markers for specific lineages, including CC19. Moreover, CC19 is associated with the horizontal transfer of macrolide and tetracycline resistance genes, thereby contributing to MDR. This highlights the significance of monitoring the spread of this clonal complex [37,66,91,92]. The absence of the globally prevalent invasive lineage (CC17) could be attributed to the population studied, as no samples from neonatal infections were analyzed. Furthermore, the study population was limited in size as it only consisted of levofloxacin-resistant strains, with only 25.6% of them being serotype III. However, studies indicated that other CCs may also become hypervirulent through capsule switching or recombination with CC17 donors [93,94]. Therefore, the examination of clonal composition is crucial for monitoring the virulence of GBS and the spread of antimicrobial resistance genes.

## 4. Materials and Methods

### 4.1. Specimen Collection

The patient population in the study consisted of individuals from whom GBS was recovered during routine diagnostics. There were no exclusion criteria for patients based on age, gender, or additional diseases. All GBS strains that exhibited resistance to levofloxacin were selected for further molecular-genetic analysis.

**Figure 5:**
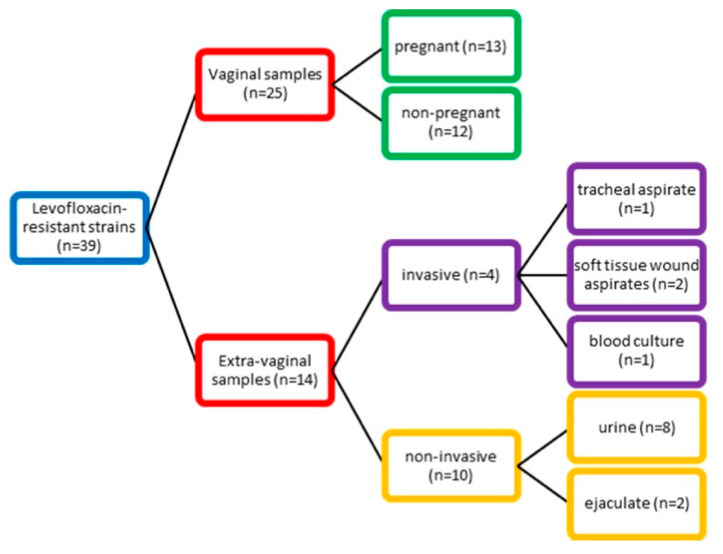
Distribution of GBS strains according to their source.

We examined 328 strains of GBS from outpatients and inpatients. The samples were collected from two university hospitals located in Sofia city (University Multiprofile Hospital for Active Treatment (UMHAT) “Aleksandrovska” and UMHAT ‘Acibadem City Clinic Tokuda’) and one hospital in Pleven city (UMHAT “Georgi Stranski”), Bulgaria, between September 2021 and June 2025. Among them, 39 were resistant to levofloxacin with an age range (AR) of 17–88 years and mean age (MA) of 42.2 years. We categorized the samples into two groups (vaginal and extra-vaginal group) according to their source (Figure 5).

### 4.2. GBS Strains

The screening methods for the presumptive identification of GBS were Gram stain morphology, negative tests for catalase and PYR, lack of susceptibility to bacitracin, positive tests for CAMP, and latex agglutination for serogroup B by Lancefield (PathoDxtra Strep Grouping Kit ThermoScientific, Oxoid, Basingstoke, UK). If necessary, subsequent biochemical identification was performed with Crystal GP (Beckton Dickinson, Kelberg, Germany).

*S. agalactiae* strains were stored at −70 °C in Skim milk BBL^®^ (Becton Dickinson, Franklin Lakes, NJ, USA) or porous beads and cryopreservative-added broth (Cryoinstant, Deltalab, Barcelona, Spain) and subcultivated three times on Columbia agar (Becton Dickinson, Kelberg, Germany) supplemented with 5.0% sheep blood for 20–24 h at 35 °C in 5.0% CO_2_ before use for antibiotic susceptibility testing and other tests. Reference strains *Streptococcus pneumoniae* ATCC 49619 and *S. agalactiae* ATCC 27956 were used to control the tests performed according to the EUCAST guidelines (2025) [95].

### 4.3. DNA Extraction

DNA extractions from pure cultures of *S. agalactiae* were performed with the DNA-Sorb A DNA Extraction Kit (Sacace Biotechnologies Srl, Como, Italy) according to the manufacturer’s instructions. All DNA extracts were stored at −70 °C.

### 4.4. Antimicrobial Susceptibility Testing

Antibiotic susceptibility testing to levofloxacin was performed by determining the MIC using the Epsilometer test (E-test) (Liofilchem, Roseti degli Abruzzi, Italy), which is defined as the “exponential gradient” method that strictly determines the antibiotic resistance of the bacteria. This method was performed on Mueller-Hinton agar with 5% defibrinated horse blood and 20 mg/L beta-NAD. The plates were then incubated at 36 °C for 18 ± 2 h. For interpretation of the results, we used the EUCAST criteria, according to which at MIC values >2 µg/l, the tested strains were considered resistant [95].

### 4.5. Serotyping and PCR Amplification of Quinolone-Resistant Genes

GBS identification, the detection of capsule serotypes, and topoisomerase gene amplification were carried out by PCR using primers previously described by Delannoy et al. (2013), Poyart et al. (2007), and Murayama et al. (2009), respectively [96,97,98,99]. For conventional PCR reaction, we used GTQ-Cycler^®^ 96 (Hain Lifescience GmbH, Hehren, Germany), prime Taq premix 2× (Genetbio Co. Ltd., Daejeon, Republic of Korea), and the results were read by gel electrophoresis using GelRed^®^ Nucleic Acid Gel Stain (Biotium, Inc., Fremont, CA, USA).

### 4.6. Automated Sequencing

The *gyrA* and *parC* PCR products were purified using the Rapid PCR Cleanup Enzyme Set (ExoSAP, Applied Biosystems, Waltham, MA, USA). Sanger bidirectional sequencing was performed by the BigDye^®^ Terminator v3.1 Cycle Sequencing Kit and BigDye^®^ Terminator v1.1 and v3.1 5X Sequencing Buffer on an Applied Biosystems 3130xl Genetic Analyzer. The obtained sequences were analyzed and compared to *S. agalactiae* GTC 1234 (susceptible to quinolones strain [26]) and *S. agalactiae* GTC 1966 (resistant to quinolones strain [26]) using the Basic Local Alignment Search Tool (BLAST) available at the National Center for Biotechnology Information (NCBI) (https://blast.ncbi.nlm.nih.gov/Blast.cgi, accessed on 3 November 2025).

### 4.7. Multi-Locus Sequence Typing (MLST)

All strains with mutations in the genes that encode topoisomerase enzymes were subjected to epidemiological typing using the MLST protocol. PCR amplification of the seven housekeeping genes in GBS: *adhP* (alcohol dehydrogenase, 704 bp)*, pheS* (phenylalanyl tRNA synthetase, 761 bp)*, atr* (amino acid transporter, 779 bp)*, glnA* (glutamine synthetase, 709 bp)*, sdhA* (serine dehydratase, 684 bp)*, glcK* (glucose kinase, 641 bp), and *tkt* (transketolase, 657 bp) was performed. The reaction was carried out using primers previously described by Jones et al. (2003) [31]. The amplification consisted of 35 cycles, commencing with an initial denaturation step at 95 °C for 30 s, followed by annealing at 54 °C for *pheS* and *glcK*, 56 °C for *glnA*, 60 °C for *atr* and *tkt*, and 61 °C for *adhP* and *sdhA*, each lasting 40 s, and an elongation phase at 72 °C for 1 min. The duration of the final elongation was 10 min. The reactions were carried out separately using a conventional PCR as aforementioned.

The PCR products obtained were subsequently purified by Exo-CIP™ Rapid PCR-Cleanup Kit (New England Biolabs, Ipswich, MA, USA). The bidirectional nucleotide sequencing was performed with sequencing primers previously described by Jones et al. (2003) through an ABI 3500 xl Genetic Analyzer (Applied Biosystems, Waltham, MA, USA) [31]. Sequence analysis was conducted using the software application FinchTV v 1.4.0. The allelic profiles and the corresponding sequence types (STs) of the isolates were determined based on the Jones scheme using a website (https://pubmlst.org). Within a single clonal complex (CC), STs exhibiting single locus variants (SLVs) or double locus variants (DLVs) were identified. All CCs were evaluated and analyzed for genetic similarity to the reference circulating clones found in the international database (https://pubmlst.org/organisms/streptococcus-agalactiae, accessed on 3 November 2025). Population structure (population snapshot) was depicted by Phyloviz 2.0 (https://www.phyloviz.net, accessed on 3 November 2025) using the goeBURST algorithm [100,101,102].

### 4.8. Statistical Analysis

Statistical analyses (serotype distribution in relation to resistance, MIC values, and CCs; CCs in relation to sample sources) were performed using IBM SPSS Statistics for Windows v19.0 (IBM Corp., Armonk, NY, USA). Fisher’s exact test was used. A *p*-value ≤ 0.05 was considered statistically significant.

## 5. Conclusions

This research investigated the population structure of levofloxacin-resistant GBS in Bulgaria and identified the genetic factors responsible for the resistance. Six distinct clonal complexes comprised of isolates from different serotypes were determined. We established a growing resistance to fluoroquinolones, which appears to exceed the previously reported European averages. More than half of the isolates exhibited mutations in both genes that encode topoisomerase enzymes, indicating a sequential, stepwise acquisition of genetic changes in the QRDRs. This situation necessitates rigorous monitoring and control to prevent the emergence of MDR GBS, along with surveillance for emerging mutations that affect the development of resistance to newer generations of quinolones.

## Figures and Tables

**Table 1:** Distribution of serotypes among levofloxacin-resistant and levofloxacin-susceptible GBS isolates.

Serotypes	Levofloxacin-Resistant (n = 39)	Levofloxacin-Susceptible (n = 289)	Total Number (n = 328)	*p*-Value * (Levofloxacin-Resistant/Levofloxacin-Susceptible)
Ia	1 (2.6%)	80 (27.7%)	81 (24.7%)	**0.0002**
Ib	0	4 (1.4%)	4 (1.2%)	
II	5 (12.8%)	42 (14.5%)	47 (14.3%)	1
III	10 (25.6%)	52 (18.0%)	62 (18.9%)	0.276
IV	5 (12.8%)	21 (7.3%)	26 (7.9%)	0.214
V	12 (30.8%)	60 (20.8%)	72 (22.0%)	0.155
VI	3 (7.7%)	0	3 (0.9%)	
VII	0	1 (0.3%)	1 (0.3%)	
VIII	0	0	0	
IX	0	0	0	
NT **	3 (7.7%)	29 (10.0%)	32 (9.8%)	0.781

* a *p*-value < 0.05 is considered statistically significant. ** NT—non-typable.

**Table 2:** Distribution of clonal complexes according to the sample source.

CCs *	Vaginal Samples (n = 25)	Extra-Vaginal Samples			Total Number (n = 39)	*p*-Value ** (Vaginal/Extra-Vaginal Samples)	Mean Age (Years Old)
		Invasive (n = 5)	Non-Invasive (n = 9)	Total Extra-Vaginal (n = 14)			
CC1	2 (8.0%)	0	2 (22.2%)	2 (14.3%)	4 (10.3%)	0.609	50.3
CC12	0	1 (20.0%)	0	1 (7.1%)	1 (2.6%)		67.0
CC19	16 (64.0%)	4 (80.0%)	5 (55.6%)	9 (64.3%)	25 (64.1%)	1	37.1
CC23	1 (4.0%)	0	0	0	1 (2.6%)		50.0
CC452	3 (12.0%)	0	0	0	3 (7.7%)		44.3
CC459	1 (4.0%)	0	1 (11.1%)	1 (7.1%)	2 (5.1%)	1	61.0
Singletons	2 (8.0%)	0	1 (11.1%)	1 (7.1%)	3 (7.7%)	1	48.7

* CCs—clonal complexes. ** a *p*-value < 0.05 is considered statistically significant.

## Data Availability

All datasets generated or analyzed during the study are included in the manuscript.

## Supplementary Materials

The following supporting information can be downloaded at: https://www.mdpi.com/article/10.3390/antibiotics14111121/s1, Table S1: Distribution of serotypes according to the MIC values; Table S2. Silent mutations in the *parC* and *gyrB* genes among levofloxacin-resistant GBS isolates; Table S3. Allelic profile and the associated STs and CCs of all analyzed levofloxacin-resistant GBS strains; Table S4: Distribution of serotypes among the most prevalent CC19 compared to other CCs.
