# Supplementary material for: Genetic Determinants and Clonal Composition of Levofloxacin-Resistant Streptococcus agalactiae Isolates from Bulgaria
Source: Antibiotics (Basel). 2025 Nov 7;14(11):1121. doi: 10.3390/antibiotics14111121 (PMC12649546; doi:10.3390/antibiotics14111121)
Supplement: Supplementary file 1 [file antibiotics-14-01121-s001.zip › antibiotics-3957496-supplementary.pdf]

Table S1. Distribution of serotypes according to the MIC values.

Serotypes	MIC>32 μg/mL** (n=23)	MIC<32 μg/mL** (n=16)	Total number (n=39)	p-value* (MIC>32 μg/mL/MIC<32 μg/mL)
IA	0	1 (6.3%)	1 (2.6%)	
II	0	5 (31.2%)	5 (12.8%)	
III	9 (39.1%)	1 (6.3%)	10 (25.6%)	**0.028**
IV	0	5 (31.2%)	5 (12.8%)	
V	11 (47.8%)	1 (6.3%)	12 (30.8%)	**0.012**
VI	1 (4.3%)	2 (12.5%)	3 (7.7%)	0.557
NT***	2 (8.7%)	1 (6.3%)	3 (7.7%)	1

* a p-value <0.05 is considered statistically significant. **MIC – minimal inhibitory concentration. ***NT – non-typable.

Table S2. Silent mutations in the *parC* and *gyrB* genes among levofloxacin-resistant GBS isolates.

	Substitution	Frequency
parC	Ala142Ala (GCG→GCC/GCA)	69.2%
	Phe145Phe (TTT→TTC)	15.4%
	His102His (CAC→CAT)	23.1%
	Ala114Ala (GCG→GCA)	15.4%
	Ile81Ile (ATC→ATT)	2.6%
	Asn161Asn (AAT→AAC)	2.6%
gyrA	Gly49Gly (GGT→ GGG)	2.6%

Ala – alanine, Phe – phenylalanine, His – histidine, Ile – isoleucine, Asn – asparagine, Gly – glycine.

Table S3. Allelic profile and the associated STs and CCs of all analyzed levofloxacin-resistant GBS strains.

Strain №	Allelic profile							ST *	CC **
	adhP	pheS	atr	glnA	sdhA	glcK	tkt		
1	1	92	3	2	2	2	2	1369	CC19
2	1	92	3	2	2	2	2	1369	CC19
3	1	92	3	2	2	2	2	1369	CC19
4	10	32	2	1	3	2	2	735	
5	5	7	6	3	2	1	3	34	CC23
6	1	92	3	2	2	2	2	1369	CC19
7	263	1	43	2	2	2	2	1324	CC19
8	263	1	43	2	2	2	2	1324	CC19
9	46	9	3	5	2	2	2	541	
10	46	1	3	2	2	2	2	233	CC19
11	1	1	3	5	2	2	2	28	CC19
12	46	1	3	2	2	2	2	233	CC19
13	1	1	3	1	22	12	2	136	CC459
14	5	25	4	2	2	3	3	1051	CC452
15	1	1	43	2	2	2	2	335	CC19
16	1	1	1	2	2	2	2	456	CC19
17	1	1	3	5	2	2	2	28	CC19
18	5	25	4	2	2	3	3	1051	CC452
19	1	74	3	2	2	2	2	959	CC19
20	1	1	3	2	75	2	2	772	CC19
21	9	1	4	1	3	2	2	1341	CC12
22	277	1	3	2	2	2	2	1661	CC19
23	277	1	3	2	2	2	2	1661	CC19
24	1	67	43	2	2	2	2	2141	CC19
25	1	1	43	2	2	2	2	335	CC19
26	1	1	43	2	2	2	2	335	CC19
27	1	1	43	2	2	2	2	335	CC19
28	36	1	2	1	1	2	2	153	CC1
29	94	1	3	1	1	2	2	645	CC1
30	1	1	3	2	2	2	7	36	CC19
31	9	8	2	1	3	2	4	2090	
32	1	1	3	94	2	2	2	908	CC19
33	1	1	2	1	1	136	2	1307	CC1
34	61	1	3	1	41	12	2	1492	CC459
35	1	99	2	1	1	2	2	1273	CC1
36	1	92	3	2	2	2	2	1369	CC19
37	5	25	4	2	2	3	3	1051	CC452
38	1	1	43	2	2	2	2	335	CC19
39	277	1	3	2	2	2	2	1661	CC19

*ST – sequence type; **CC – clonal complex.

Table S4. Distribution of serotypes among the most prevalent CC19 compared to other CCs.

Serotypes	CC19 (n=25)	Other CCs** and singletons (n=14)	Total number (n=39)	p-value* (CC19/other CCs and singletons)
IA	0	1 (7.1%)	1 (2.6%)	
II	2 (8.0%)	3 (21.4%)	5 (12.8%)	0.329
III	9 (36.0%)	1 (7.1%)	10 (25.6%)	0.064
IV	0	5 (35.7%)	5 (12.8%)	
V	10 (40.0%)	2 (14.3%)	12 (30.8%)	0.151
VI	2 (8.0%)	1 (7.1%)	3 (7.7%)	1
NT***	2 (8.0%)	1 (7.1%)	3 (7.7%)	1

* a p-value <0.05 is considered statistically significant. ** CC1, CC12, CC23, CC452, and CC459. ***NT – non-typable.
